# Rare brain metastasis with unusual characteristics in a late recurrence of stage IIIA uterine papillary serous carcinoma

**DOI:** 10.1259/bjrcr.20200157

**Published:** 2021-03-18

**Authors:** Charles Nicolas Crain, Remy Ngwanyam, Gregory Punch

**Affiliations:** 1 Department of Radiology, Tripler Army Medical Center, Honolulu, HI, USA

## Abstract

Uterine papillary serous carcinoma (UPSC) is a rare endometrial neoplasm with high mortality rates. While the malignancy has often metastasized to distant organs by the time of diagnosis, brain lesions are extremely rare and most commonly only observed in widely disseminated disease. Here, we present an unusual case of UPSC with brain metastasis discovered six years after undergoing treatment for stage IIIA disease. Compared to the few previous cases of brain metastasis from UPSC, this lesion exhibited unusual imaging characteristics. We also highlight a potential imaging interpretation pitfall which was associated with this case.

## Case presentation

A 57-year-old female presented to the Emergency Department with blurry vision and slight vertical diplopia that developed gradually over several days. Her history was notable for FIGO stage IIIA uterine papillary serous carcinoma (UPSC) treated with total abdominal hysterectomy, bilateral salpingo-oophorectomy, chemotherapy, and radiation therapy six years earlier. She had since been stable and received regular biannual follow-up with gynecological oncology with no evidence of recurrence. She had no other major medical problems and took no medications. Physical exam was unremarkable except for visual acuity of 20/50 OU, OS, and OD. Neurological exam was normal without evidence of cranial nerve palsies.

## Imaging

**Figure 1.**
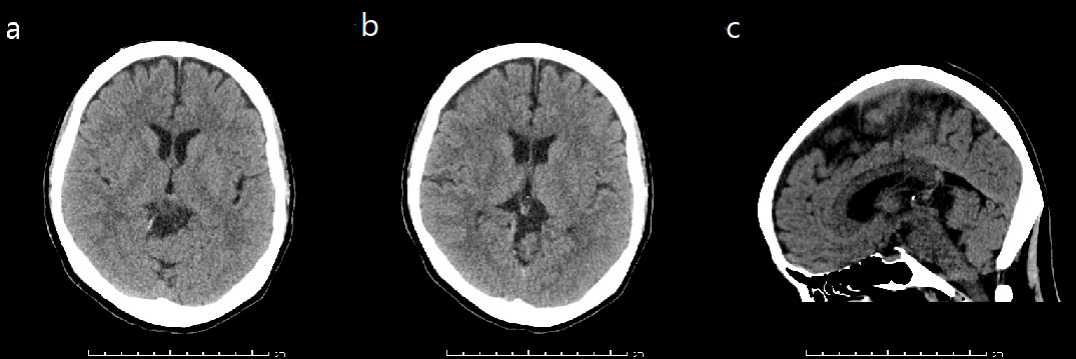
A non-contrast head CT reveals an extra-axial hypodense mass within the quadrigeminal cistern measuring approximately 1.5 × 1.2 × 0.9 cm. Mild mass effect upon the tectum and anterosuperior cerebellum was observed. Shown above are axial (**a, b**) and sagittal (**c**) views of the lesion. No comparison images were available at the time.

A non-contrast head CT revealed an extra-axial hypodense mass within the quadrigeminal cistern measuring approximately 1.5 × 1.2 × 0.9 cm with mild mass effect upon the tectum and anterosuperior cerebellum (Figure 1).

**Figure 2.**
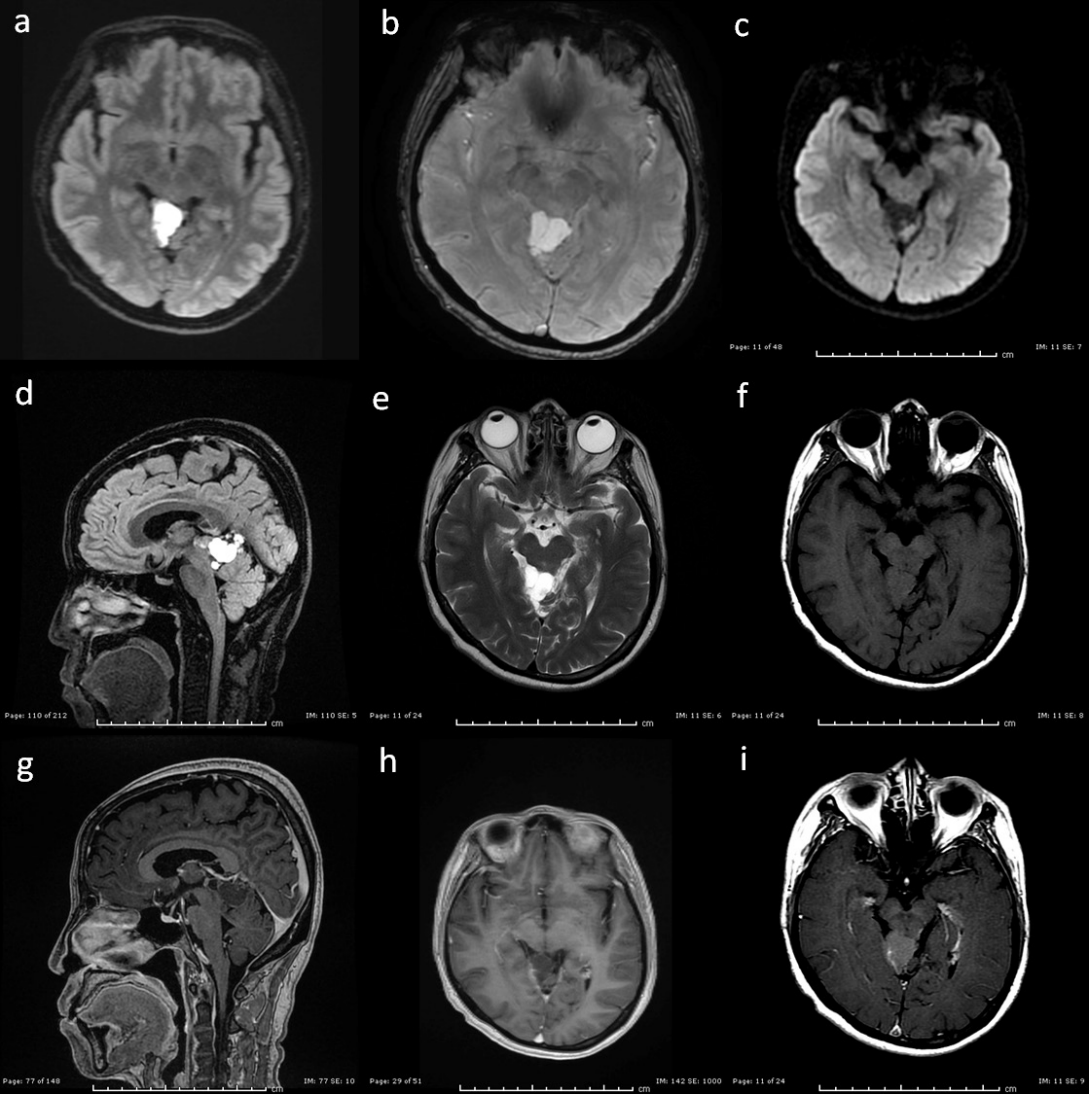
Axial FLAIR (**a**), axial SWI (**b**), axial DWI (**c**), sagittal FLAIR (**d**), axial T2 (**e**), axial T1 SE (**f**), sagittal 3D FSPGR post (**g**), axial 3D FSPGR post (**h**), and axial T1 SE Post (**i**). Multiplanar pre- and post-contrast images of the brain demonstrate an extra-axial lobular mass in the supracerebellar and quadrigeminal plate cisterns, causing mild mass effect on the brainstem and superior cerebellar vermis without signs of obstruction or adjacent parenchymal edema or gliosis. The lesion demonstrates FLAIR and T2 hyperintense signal characteristics (**a,d,e**), with intermediate heterogeneous T1 signal intensity (**f**) without restricted diffusion (**c**) or internal susceptibility signal (**b**). 3D gradient T1W post-contrast images (**g,h**) demonstrate no significant enhancement whereas conventional axial post-contrast image (**i**) shows mild, heterogeneous enhancement.

Subsequent brain MRI with and without gadolinium contrast was significant for a multilobular, heterogeneous, primarily hyperinstense T2 lesion within the quadrigeminal cistern with extension into the ambient cisterns (Figure 2). The lesion measured approximately 3.0 × 2.0 × 2.0 cm. Heterogeneous T1 and FLAIR signal was present. Of note, fast spoiled gradient-echo (FSPGR) post-contrast images demonstrated no significant enhancement, whereas conventional spin-echo axial post-contrast image showed mild, heterogeneous enhancement. No reduced diffusion signal or internal blood products were demonstrated. There was mild mass effect on the dorsal midbrain as well as the cerebellar vermis without any evidence of gliosis of the underlying cerebellum.

## Differential diagnosis

Differential diagnosis for intracranial extra-axial lesions may include neoplasms, developmental cysts, and infectious/inflammatory cause like sarcoid, hemorrhages, and vascular abnormalities. The mass did not demonstrate blooming artefact on the SWI sequence, which ruled out hemorrhage. Appearance and location would also be unusual for an extra-axial bleed. While conventional T1W post-contrast images demonstrated mild heterogeneous enhancement, it was very subtle and the lack of significant enhancement on FSPGR images or pulsation artefact ruled out possible aneurysms or other vascular malformations. The lesion did not follow CSF signal on all sequences, which ruled out an arachnoid cyst. A dermoid cyst was considered although less likely as the mass demonstrated intermediate T1 signal without the typical T1-shortening seen in fat-containing lesions. Metastatic disease was initially thought less likely given lack of significant enhancement, unusual location and the fact that it was a solitary lesion. An atypical epidermoid cyst was suggested as the most likely diagnosis given incomplete signal drop out on FLAIR sequences and lack of true restricted diffusion. Neurosurgery was recommended for tissue diagnosis given absent characteristic imaging findings for a specific mass as well as the patient’s vision loss.

## Treatment

The patient underwent craniotomy with near gross total resection of the lesion. Pathology results indicated a papillary carcinoma, favoring metastatic UPSC given its immunoreactivity to PAX8 and her clinical history. She was referred to gynecologic-oncology, medical oncology, and radiation oncology. Six weeks after surgery, brain MRI revealed the residual small septated cystic mass centered in the quadrigeminal cistern and left ambient cistern. After subsequent PET scan did not reveal evidence of disease elsewhere, decision was made to treat with radiation therapy and chemotherapy was deferred.

## Outcome and follow-up

The patient tolerated radiation therapy well which resulted in interval reduction of her intracranial mass. She is on a surveillance plan of annual PET scans and head/spinal MRI’s every 3–4 months. She also receives follow-up with gynecologic oncology, radiation oncology, and neurosurgery every 3–4 months and is overall doing well seven months after her diagnosis was confirmed by pathology.

## Discussion

While only accounting for 10% of endometrial cancers, UPSC accounts for 39% of its deaths.^1^ It has a higher rate of abdominal and systemic recurrence and lower rates of survival when compared to endometrioid endometrial carcinomas (EEC), the most common form of cancer in the female reproductive tract.^2,3^ Brain metastasis from endometrial cancer is extremely rare, accounting for 0.6% of cases from a review of over 10 000 patients.^4^ A review by Sierra et al^5^ in 2015 identified nine cases of UPSC that elicited brain metastasis, a 2015 case series by Narasimhulu et al^6^ described two more and a 2019 case report by Filho et al^7^ described another. This appears to be the thirteenth that has been reported.

This patient’s case was notable for its long interval from primary diagnosis to identifying brain metastasis. Only one other case out of the thirteen exhibited this interval greater than five years.^5,8^ Also unusual was her brain mass being the solitary metastatic lesion. Metastatic UPSC is usually found in the brain only in widely disseminated disease, and only four of the other twelve cases exhibited this characteristic.^4,5,9,10^ Finally, this patient is notable for her relatively successful clinical outcome for a highly malignant form of endometrial carcinoma. Average survival after diagnosis of a brain metastatic lesion from UPSC was found to average five months in the first nine reported cases by Sierra et al. and only one of the other eleven cases was alive at the time of case write-up.^5^ At the time of this case’s submission, it has been 14 months following her initial CT scan and she continues to do well.

The imaging findings were notable for several reasons. First, it appears to be the first reported lesion in the quadrigeminal cistern. Of the eight prior cases in which location was described, lesions were intra-axial and found in the frontal, occipital, temporal, and parietal, regions as well as the cerebellum.^5^ The cases varied between solitary and multiple brain lesions. Second, this case appears to be the first reported to not display a strong element of post-contrast enhancement. As described above, while conventional post-contrast images did display mild heterogeneous enhancement, FSPGR images did not enhance significantly. Contrast enhancement was noted in cases presented by Narasimhulu et al^6^, Filho et al^7^, Gulsen et al^11^, and Sierra et al^5^. Finally, the mass in this case had a heterogeneous appearance on all sequences, differing from cases presented in Filho et al^7^, Sierra et al^5^, Narasimhulu et al^6^, and Gulsen et al^11^, which were homogeneous.

Chappel et al., Furutani et al., and others described that interpretation of images obtained by gradient-echo sequences has the potential to miss findings more easily visualized with spin-echo sequences.^12,13^ This was evident in our case as shown in the images above. There was no significant enhancement shown in our FSPGR images, while our conventional spin-echo images showed mild heterogeneous enhancement. This serves as a reminder that although there are advantages to gradient-echo sequences, spin-echo is preferred for capturing the full spectrum of contrast-enhancing lesions within the central nervous system.^12^ Thin slices obtained by 3DFSPGR sequences can mitigate this potential pitfall.^13^

The explanation for the mass’s unusual location and imaging characteristics is unclear. Given the low sample size of cases demonstrating metastasis to the brain, it is difficult to confidently describe what the characteristic imaging appearance should be. The difference in presentation from the prior cases only adds to our general knowledge and should heighten our awareness of the variability of presentation as opposed to the previously reported cases of an intra-axial, strongly enhancing homogenous mass.

## Learning points

1. Uterine papillary serous carcinoma (UPSC) accounts for only 10% of endometrial cancers but 39% of its deaths.
2. Endometrial cancer with brain metastasis is extremely rare, occurring in only 0.6% of cases. This patient appears to be the thirteenth reported case demonstrating the rare phenomenon of UPSC metastasizing to the brain.
3. Based on the limited data from previous cases, metastatic UPSC to the brain typically presents as an intra-axial lesion has a homogeneous appearance on all MRI sequences, and normally strongly enhances with contrast administration. This case was unusual in that it differed in each of these characteristics.
4. Spin-echo is normally superior to gradient-echo in detecting contrast-enhancing lesions, despite the advantages of gradient-echo sequences. Thin slices of 3DFSPGR sequences mitigate this potential pitfall.
